# Abstracts and Gleanings

**Published:** 1877-02-20

**Authors:** 


### Abstracts and Gleanings.

*Treatment of Diphtheria.*—"I will not stop to enumerate the long list of remedies used, but will confine myself to the method which I have adopted, and with such evident success that I feel glad to announce to any of you who have not followed the same line of treatment that you will be compelled to say—'Eureka.' I am sure I feel quite as enthusiastic in the success of the treatment which I propose to lay down as one of our number is in the treatment of variola with milk-punch and egg-nog. If you are permitted to see the patient within the first few hours of the attack, commence your treatment at once with quinine and aromatic sulphuric acid in doses suitable to the age of the person receiving it. Give freely of solution of chlorate of potassa, as a disinfectant, and perhaps you will not be required to administer any other remedies. If, however, the membrane has become so thickly deposited as not to be affected by the acid and chlorine, you should apply with your own hand a mop, properly made, saturated with the liquid persulphate of iron, and literally swab out the throat until you remove every particle of membrane. Let this be repeated two or more times each day, or as often as the membrane would continue so to be reproduced, and you will have the satisfaction of seeing your patient make a speedy recovery without any of the consequent sequelæ. I took my first hint of the sulphuric-acid treatment from a short extract which I clipped from a paper coming from a doctor in Australia, where the disease was producing such extensive ravages that the government offered a large reward for any certain method of cure. I will quote from the paper: 'It is simply the use of sulphuric acid, of which four drops are diluted in three fourths of a tumbler of water to be administered to a grown person, and a smaller dose to children, at intervals not specified. The result is said to be a coagulation of the diphtheritic membrane, and its ready removal by coughing. It is asserted, where the case thus treated has not advanced to a nearly fatal termination, the patient recovered in almost every instance.' This suggested to me the treatment which I have already announced; and from the experience of entire success which I have had in the last two years in not having one fatal case during that time from that disease, where I had the treating of the case from the beginning, I do not hesitate in declaring it as my opinion that quinine as an eliminator of the poison from the system, and sulphuric acid as a detergent to the throat, are decidedly as much a specific for diphtheria as quinine is for intermittent fever, or iodide of potash and bichloride of mercury are for tertiary syphilis."—*Phil. Med. Times.*

---

*Differences Between the Anæsthesia of Ether and Chloroform.*—Prof. Schiff (*Brit. Med. Jour.*, May 22, 1875), relates the results of more than five thousand experiments on the differences between ether and chloroform anæsthesia. With both, paralysis of conscious sensation, paralysis of the movement of voluntary muscles, paralysis of respiration, circulation, and, finally, paralysis of the heart and the vaso-motor nerves, occurs. Respiratory paralysis is produced by ether when circulation and blood pressure remain within the limits compatible with life. Sometimes the vascular pressure increases, sometimes it decreases, but is always sufficiently high to allow the exchange of the carbonic acid gas with the oxygen of the atmosphere. Vascular succeeds respiratory paralysis when ether is administered. The reverse takes place with chloroform. Frequently an amount of this anæsthetic agent which would not be sufficient to produce respiratory, may suffice to bring on vascular paralysis. Under these conditions, and when vascular paralysis lasts under thirty seconds, artificial respiration is useless, because there is no longer any exchange of gases, the blood pressure being diminished. The cessation of respiration, therefore, is not the most dangerous moment to animal life when etherization is employed, whilst it may be so with chloroformization, because sometimes it is possible to produce some automatic respiratory movements; but, nevertheless, respiration ceases immediately and the animal dies. With etherization, on the contrary, when some automatic inspirations are obtained it may be taken as certain that respiration will continue, and that the animal will live. Schiff affirms that in the present state of our science there are no means which will show us how to recognize, so as to prevent them, the tendencies which may cause death in some animals after the first inhalations of chloroform before having produced true anæsthesia. The reverse occurs with ether, so that it may be said that, in the present state of knowledge, the surgeon is responsible for the death of the individual by etherization, whilst he is not responsible when death occurs from chloroformization. From these facts Schiff concludes:

1. The phenomena relating to the paralysis of sensibility and movement are common to both ether and chloroform.

2. The two other orders of phenomena—that is to say, those relating to vascular and respiratory paralysis—often show themselves in inverse order with reference to these two agents.

3. With chloroform, however, either the one or the other of these two paralysis may first show itself, involving great danger to the animal if the vascular phenomena be the first to make their appearance. Therefore the use of chloroform should be rejected and ether only used.—*Jour. of Mat. Med.*

---

*Gelseminum as an Antidote to Veratrum Viride.*—Gelseminum has been successfully used in a case of poisoning by veratrum viride (*Journal of Materia Medica*, N. Y.). The following is recommended by Dr. Wilson:—Five drops of fluid extract of gelseminum, with a quarter of a grain of powdered nux vomica, administered every fifteen minutes.—*The Doctor*.

[We have never yet been able to ascertain what is a poisonous dose of veratrum viride. We have seen it administered in teaspoonful doses of Norwood's tincture, and we have habitually given from ten to thirty drops of the same tincture every two hours without producing any symptoms more alarming than those witnessed after taking half a grain or so of tarter emetic; and these symptoms invariably cease in an hour or two if the medicine is discontinued. We very strongly suspect, therefore, that the supposed antidotal effects of gelseminum alluded to above, were due to *time* alone and not to the medicine. There is no single medicine capable of antagonizing veratrum, because the effects of the latter are twofold. First, it is a vascular sedative, and secondly (but not invariably), a nauseant: and it is wrong to infer that the first is consequent to the second; for we are in the constant habit of obtaining the first effect without producing any nausea at all, by simply combining the medicine with morphia or tincture of opium. For the nauseating effect of the drug, therefore, opium or one of its preparations is a perfect antidote; while for its effects upon the heart, alcohol in some form is probably the only—certainly an all-sufficient—antagonist. Certainly, if the generally received views of the physiological action of veratrum and gelseminum are correct, the latter is directly contra-indicated in the so-called veratrum poisoning.—*Ed. Phys. & Surg.*

---

*Cool Bathing in the Treatment of Inflammatory Bowel Affections During the Summer.*—I beg to offer a method of treatment of bowel affections of children in the summer season, where fever is present. It has been so successful with me, that I am confident, if applied more generally, it would lessen very greatly the rates of mortality in the summer season. I allude to that form of disease which is denominated entero-colitis.

Before we are called to these cases, tentative measures for the relief of the diarrhœa have already been applied by the friends, so that the inflammatory stage is generally fully developed when we first see the patient. The skin is hot (temperature 102½° to 105°), the pulse rapid (130 to 150), respiration 30 to 50, with frequent purging of semi-fluid, greenish watery, fecal, and half digested matters; the mouth and tongue are dry; the thirst intense, but the water taken to slake it is quickly thrown off; the eyes are staring; pupils contracted; insomnia and rolling of the head, with utterance of distressing cries, due to headache from hyperæmia of cerebral vessels and unappeased thirst. Such is a general statement of the symptoms.

I at once proceed to give the little sufferer a bath in hydrant water, which with us, in summer, is about 75°. I have found it necessary to give this my personal attention at first, the mother or friends will not carry out instructions, on account of the cries and resistance of the child; it seems to them a great cruelty.

The contact of a hot skin with cold water is certainly painful for the moment, hence I immerse the body from legs upward gradually, sponging the skin in advance, so as to obtain tolerance.

When the body and extremities are fully under, holding the head in the palm of my left hand, I pour over its surface cooler water, such as cistern water, which is here about 65°. This is kept up for ten or fifteen minutes. Meanwhile the child ceases to cry or struggle, and is evidently greatly comforted; more especially when cool water is freely given to drink—the greedy swallowing of which shows how much of its distress is due to thirst.

After the bath the patient should be wrapped, unwiped, in a light woollen shawl, and laid upon its bed, with a slight additional covering. The pulse has lost frequency, but is quite feeble the breathing is slower and the skin quite cool, even bluish in hue. The sedation may seem at first too great; but reaction soon begins, a healthy warmth and perspiration are established, and the child falls into a peaceful sleep. The scene has so changed that one will find no difficulty thenceforth in getting a bath given three or four times in twenty-four hours, if the alarming train of symptoms make show of revival; and they will revive to such an extent as to require exhibitions of the bath from time to time for two or three days perhaps; for the diseased state of the mucous membrane within has not been as suddenly relieved as the abnormal heat of the body.

In the meantime internal remedies should be freely employed. Quinine, whisky, beef-tea, milk, and lime-water are the chief agents. One grain of quinine and a drachm of whisky every three hours, for a child eight or sixteen months old, looks rather formidable, but they will be found admirable while the disposition of fever lasts.

Subsequently bismuth and pepsin are of great value to restrain diarrhœa and to assist digestion, so greatly at fault owing to the blow which the mucous membrane has suffered.—O. G. Comegys.—*Medical Record.*

---

*Bromide of Arsenic in the Treatment of Epilepsy.*—Dr. Th. Clemens, of Frankfort-on-the-Main, has employed bromide of arsenic for twenty years in the treatment of diseases of the nervous system, and especially of epilepsy, and claims that he has obtained astonishing results with it. He uses the liquor arsenic. bromat., and gives one or two drops in a glass of water once, or, if necessary, twice daily. These minute doses may be given for months and even years, without producing the usual unpleasant effects of a long-continued arsenical course. All his cases of epilepsy have been markedly relieved and improved by this remedy, but in only two cases has it produced a complete cure. In many cases of incurable epilepsy, complicated with idiocy and deformities of the skull, the fits were reduced in number from twenty in the twenty four hours, to four or even two, a result that has been obtained by no other treatment. In connection with the bromide of arsenic, an almost exclusively meat diet is advised. The patients should be as much as possible in the open air in the daytime, and their windows be kept open at night. Unlike bromide of potassium, this remedy does not require to be given in increasing doses, and instead of interfering with digestion, improves the nutrition and strength. Dr. Clemens has employed the following formulæ since 1859, and thinks that it ought to replace Fowler's solution, which is irrational in its composition and uncertain in its action. This solution becomes stronger with time; the chemical union of the bromide with the arseniate of potash becoming more and more perfect.—R Pulv. Arsenic. alb., Potassa. carb. e. tartar., aa dr. i.; coque cum aqua destil. lb. ss. ad solut. perfect.; adde, aq. evaporat. restituta, aquæ distil. oz. xij., dein adde brom. pur. dr. ij., refrigerat. stet per sufficient. temp. ad decol., S. liq. arsenic. bromat.—*Allg. Med. Central-Zeitung*, May 24th.

---

*Treatment of Syphylis.*—A writer in the New Orleans *Medical Journal* gives the following:

Five cases are under observation at the present time, some eight months having elapsed since infection in two instances, and fully a year in the other cases. The patients are all situated under favorable hygienic circumstances, and have at no time been unfitted for social life or attendance upon business. A slight roseola upon the abdomen, chest, and arms, preceded by slight febrile action, a few superficial mucous patches upon the lips and tonsils, make up the sum total of the lesions which have been observed. The treatment has consisted in small doses of the protiodide of mercury, one-third of a grain, thrice daily, with inunctions thrice weekly with the 10 per cent. oleate of mercury, and the most scrupulous preservation of the general health by means of tonics and attention to hygienic measures. This plan of treatment, known as the *eliminative*, has recently been ably presented to the profession in this country by Drs. Van Buren and Keyes, of New York, and seems worthy of the highest favor. From our very limited experience quite an abiding faith has been established in the statement of these gentlemen that a patient of good general health and temperate habits, under favorable hygienic surroundings, and treated from the beginning for a period of two years in this manner with mercury, followed up by a year's use of mercury and iodide of potassium in combination, will have no serious lesion, and will continue to enjoy as good health afterward as before the attack.

---

*Rabies Successfully Treated by Woorara.* The *Veterinary Journal* for July states that Offenberg, in his recent inaugural dissertation at the Berlin Hospital, gives the case of a girl, twenty-four years of age, who had been bitten eighty days before by a dog supposed to be rabid. Injections of morphine and the inhalation of chloroform having been used without benefit, seven injections of woorara, amounting in all to three grains, were given in the course of five and a half hours. First the muscles became steadier, then the convulsive seizures less frequent, the dread of water and the photophobia disappeared and the voice improved. Some symptoms of paralysis now appeared, which reached their maximum the following day. On the second day there was a slight relapse of the symptoms of rabies, which was checked by the injection of a little less than half a grain of the woorara. The patient recovered slowly, a certain degree of weakness and sluggishness, and especially weakness of sight, remaining at the end of two months. There was inflammation, but no suppuration, at the points of injection.—*Medical Press and Circular*, July 12, 1876.

---

*Treatment of Certain Forms of Acne.*—Dr. Chantry claims that he has obtained gratifying success in the treatment of rebellious cases of acne of the tuberculous and hypertrophic variety, by the use of iodide of sulphur internally in combination with Hardy's lotion externally. He gives at first one, then two or three of the following pills: R. Sulphur, iodide, gr. ss.; Extr. solani dulcamaræ, gr. ij. M. He employs also the following lotion: R. Aquæ, oz. iijss.; Tr. benzoin, dr. i.; Potass. sulphuret, dr. i. M. A teaspoonful in lukewarm water, to be used morning and evening. (Hardy.) If this lotion causes too much irritation, it must be replaced by lotions of filtered bran-water. In some cases the iodide of sulphur causes gastralgia, and its use must be discontinued; but if this does not occur, a noticeable amelioration of the affection is found in about twelve to twenty days. The hard, purple elevations which surround the tubercles slowly soften and become less swollen, the usual desquamation of the epidermis takes place, and soon nothing remains but a diffuse, pale congestion, which disappears slowly, and is often succeeded by triangular cicatrices.

In a case of acne rosacea of the face, of nine months' duration, which had resisted several methods of treatment, the iodide of sulphur could not be borne, and iodide of potassium was given instead, in doses rapidly increasing to a dracmh a day. At the same time the diseased parts were rubbed briskly every evening with a sulphur pomade (15 sulphur to 30 lard). In fifteen days the cure was almost complete, and two months later there had been no return of the disease.—*Lyon Medical*, June 18.

*Treatment of Typhoid Fever by Ergot.*—At the meeting of the *Association Francaise pour l'avancement des Sciences*, of August 23d, M. Deboue, of Pau, stated that he had treated a number of cases of typhoid fever with ergot, and that his success has been satisfactory. The toleration of ergot increases with the severity of the disease. As a rule, the drug is not so well tolerated by women as by men; consequently it must be given in smaller doses to the former. It may be given without fear to pregnant women. The pulverized ergot of rye preserves all its medical qualities for about eight days; if it lose its physiological properties within that time, it is because it was already altered when pulverized. Of fifteen cases treated by Dr. Duboue, the extreme rapidity of the cure rendered the diagnosis of two uncertain; five cases of moderate gravity that recovered presented during their courses alternations of aggravation and amelioration that corresponded with intentional interruptions of the treatment. Of eight very grave cases, six recovered; three of these cases being already far advanced before the ergot treatment was begun. In the two fatal cases, the ergot did not produce its ordinary therapeutic effects, and on examination it was found to be worm eaten and covered with a grayish powder.—*Gazette Hebdom. de Med. et de Chir.*, September 1st.

*The Cold Douche and Friction in the Treatment of Consumption.*—Sokolowski has published (*Berliner Klin. Woch.*, No. 39 and 40) a series of articles showing the value of the cold douche with subsequent friction in the treatment of consumption. He makes a summary of the indications for their use as follows:

I. Principally in individuals who have a well-marked tendency to consumption, provided there is a good general condition with a sufficient reaction after the douche.

(*a*) Individuals born of consumptive parents who are at the age of rapid growth even though there be no appearance of the disease.

(*b*) Persons who have extreme sensitiveness of the mucous membrane of the respiratory passage, *i. e.,* who easily "take cold."

(*c*) Those who have catarrh of the apices (spitzen-catarrh).

(*d*) In case of chronic bronchitis having no definite localization, and which are dependent on hereditary tendency.

(*e*) Anæmia from hereditary causes.

II. In patients who already have consumption the douche is indicated.

(*a*) In all cases of inflammatory (acquired) phthisis, provided there is a good constitutional condition.

(*b*) In constitutional (hereditary) phthisis, in case the destructive processes are limited, and the general condition is good.

In the winter season the douche should be used with great circumspection, and only in patients who are well nourished and strong.

*Death from Chloroform, in which all the Usual Precautions were Taken to Guard Against It.*—A man, aged thirty-three years, was admitted into the Charing Cross Hospital (London), Dec. 20, with a recent and irreducible, although not strangulated, hernia. After the usual efforts at reduction in the warm bath, etc., the administration of chloroform was advised. Dec. 22. There had been no vomiting. From the time of his admission until his death the patient was kept upon liquid diet. *The Brit. Med. Journ.* (December 23, 1876), in giving the details of the mode of death, says: On Friday, he had his half-pint of beef-tea at 1 o'clock, and was ordered to have no more food after that hour, as the chloroform administration was fixed for the evening. The man lay quietly in bed, and did not complain of exhaustion, but asked to have as little chloroform as might be, saying that he was not a strong man. He had been a publican, but there is no evidence as to his habits. Shortly after 8 o'clock, the administration was commenced by one of the assistant resident medical officers, who was quite accustomed to the duty. The patient lay in bed, loosely clad. The heart was examined by the stethoscope, and considered to be normal, and no stimulant was administered. The anæsthetic was given on folded lint, one-half drachm being poured on at a time. At the end of four or five minutes, when the fifth half drachm had been poured out, the taxis not having been commenced, and there having been no marked struggling, the face suddenly became livid; and the administrator, having his finger on the pulse, noticed it to become feeble, at the same time that respiration became slightly stertorous. The chloroform was at once removed, a pillow was placed under the patient's shoulders, so that the head fell rather backwards, the tongue was drawn forward by forceps, and artificial respiration, by Sylvester's method, was commenced. As soon as possible, two ounces of brandy, with warm water, were injected into the rectum; the chest was also flapped with wet towels, and the extremities rubbed and warmed. The patient, however, gave no sign of rallying, and ceased to breathe in the course of three or four minutes from his seizure. Later on, faradism was applied to the phrenic nerve, and artificial respiration was continued for an hour, but without result. At the post-mortem examination, the lungs and brain were found congested. The heart was large, and its right cavities were full of dark blood. The right ventricle was noted to be thinner than normal, and was overlaid with fat. There was no naked-eye evidence of fatty degeneration, and the microscope was not used. The liver was fatty. An inquest was held, and a verdict returned to the effect "that deceased died from the effects of chloroform, but that it was properly administered."

---

*Varicocele—Six Cases Treated by One Method.*—We saw seven cases of varicocele, in five of which the disease was confined to the left side, and in the remaining two it was developed upon both sides. Six of those patients were under treatment by means of mechanical pressure afforded by a truss, and this was aided by a suspensory bandage. The odd case, the disease being only slightly developed, was treated by means of the suspensory bandage alone. Support the pendant parts, and at the same time make moderate compression immediately over the external abdominal ring. To make pressure, an ordinary hernia truss was used, with the addition of a perineal band to secure it perfectly in position. The aim was to make such an amount of pressure as would moderately compress the veins at that point, and maintain it night and day, the truss being removed only for purposes of cleanliness. It was believed by the visiting surgeon that we should not resort to any more violent means of cure in a majority of cases, and that in a large proportion favorable results might be expected.

*Propylamine.*—"Thus far almost the only application made of trimethylamine is in the treatment of *acute rheumatism* and *gout.* In some cases it appears to produce almost complete relief after the administration of a few doses, but generally a longer time is required (Awenarius, Dujardin-Beaumetz, Spencer, Leo). It moderates at once, the fever and the joint-pain, and very decidedly shortens the duration of the disease. It is said to diminish the tendency to cardiac complication.

"This agent, having so decided an influence on the pulse, temperature, and excretion of urea, will in the future, doubtless, be applied to the treatment of other diseases.

"Chemically trimethylamine is incompatible with the mineral acids, the salts of the metals, the alkalies (chlorides), and vegetable infusions. It should always be prescribed alone, in solution, in some aromatic water. Therapeutically, it is antagonized by the stimulants, opium, belladonna, digitalis, etc.

"For therapeutical purposes, the chloride, which is specially commended by Dujardin-Beaumetz, has the advantage of being free from the disagreeable taste of propylamine. It is almost odorless, and in solution has an alkaline but not unpleasant taste. The ordinary dose is two grains every three hours."

*The Antagonism between Strychnia and Monobromide of Camphor.*—The researches of Dr. Valenti Y. Vivo with these drugs are highly interesting, and his conclusions (*Veternary Journal*, July, 1875) are worthy of being placed on record. They are:

1. The bromide reduces the force and frequency of the tetanic convulsions produced by strychnia, the tonic convulsions being converted into atonic, and the action of the antidote is rapid and sure. A strong dose of the bromide is required.

2. The bromide acts on the sympathetic, causing myosis and cardiac paralysis.

3. It is preferable to introduce the bromide by gastric ingestion, and in small and repeated doses.

4. That from four to six grammes of the bromide in small doses should be given in cases of poisoning by strychnia.

5. Twelve dogs after receiving a fatal dose of strychnia were saved by monobromide of camphor.—*The Doctor.*

*Inhalation of Iodine.*—Dr. Seguin remarks:—"I beg leave to say, also, that for more than fifteen years, I usually prescribe the inhalation of iodine in forms whose formulary may be found in many drug stores in this city. The most usual of these forms being that of a pillow containing aromatic plants, say seaweeds, black walnut or fern leaves, etc., according to secondary indications. In this pillow is introduced a little bag or satchel containing a drachm or so of iodine, in as much of bran as will prevent the too rapid evaporation of the drug. When the satchel does no more smell of iodine, it is refilled, and when the pillow begins to smell the pus-like odor peculiar to those cases, the herbs are also renewed. Let us remark *en passant* that the alteration of both is in proportion to the gravity of the affection. The pillow must be soft, and broad enough for the head and chest to remain upon it during the night tossings. The urine has to be tested for albumen during this treatment." — *Medical Record.*

*Gastric Ulcer—Milk Treatment.*—A female patient suffering with this disease was admitted, and at once placed upon such quantities of milk as could be taken without being rejected by the stomach. The amount given at first was only a teaspoonful, which could be repeated about every twenty minutes. The milk was increased in quantity only as it could be tolerated by the stomach, and the patient was then able to take half a glass at a time. In addition a piece of cardboard about the size of a five-cent nickel was dipped in nitric acid, and laid over the region of the tenderness in the epigastrium, and permitted to remain until the cuticle was destroyed, when it was removed and the sore kept open by dressing it with a piece of adhesive plaster. The patient had been in the hospital several times suffering from the vomiting, etc., incident to this affection, and had usually been treated with injections. But her recovery at this visit had been equally prompt and much more agreeable, although her symptoms had been as severe as at any previous admission.—*Medical Record.*

*Citrate of Soda in Diabetes.*—M. Guyot Darmecy recommends "Citrate of Soda" in daily doses of half a drachm to one drachm, as an excellent remedy in this disease. It has been shown by analysis, that sugar disappears from the urine when this salt is used with the food instead of common salt. It is also known, since the researches of Whœler, that the alkaline salts of organic acids, when given in doses too small to produce purgative effect, absorbed, and their acid being burnt up in the respiartory process, are elimanated by the urine as carbonates. Hence, "Citrate of Soda" may, without interfering with the gastric acid in the same way as alkaline carbonates, place the system under the influence of an alkaline carbonate, which is indispensable to the interstitial combustion of the glucose of the food.—*Medical Brief*, July, 1876.

*Forcible Dilatation of the Sphincter Ani in the Treatment of Hemorrhoids.*—Dr. Christofari in his work on this subject comes to the following conclusions:—Contraction of the sphincter ani plays an important role in the production of hemorrhoids. This contraction is much more frequent in hemorrhoidal affections than most writers on the subject think. It produces the constipation and the acute pain which these patients suffer before and after defecation. It exists, too, without pain, and for this should be none the less considered as a cause productive of hemorrhoids. In removing the constriction the physician relieves the painful symptoms which it occasions and at the same time may treat the diseased parts. The best method to employ is that of forcible dilatation. This proceeding, so simple and so harmless, has succeeded in a very great number of cases.—*Gaz. des Hopitaux.*

*A Prophylactic for Sore Nipples.*—Dr. Julius Fehr writes (*New York Medical Record*, August 21st):—The curative, as well as the palliative, treatment of sore or cracked nipples being well known to be futile, my aim for a long time was directed to the finding of a reliable prophylactic. After trying a good many formulas of others, and combinations of my own, I came at last to the use of tannate of lead, the "cataplasma ad decubitum" of the *Pharmacopœia Germanica* with the addition of a little glycerine to modify, in some degree, the excessive drying properties of that preparation, This "plumbum tannicum pultiforme" I had applied, for about one month before parturition, two or three times a day, directly to the nipples. This I found "tanned" the nipples in so thorough a manner that they were perfectly able to withstand all suckling, and all pulling on the part of the infant successfully. At the same time, I use a piece of cotton felt, about one inch and a half in diameter, and half an inch in thickness, with an aperture in the middle large enough to give free access to the nipple. This will not only prevent the pressure of the garments on the nipples, but will give, at the same time, to the nipples a chance to develop themselves better, which is often so much needed.—*Brit. Med. Jour.*

*Atropia Poisoning.—Dr. Leonard.*—Assisted by Dr. McDowell I operated for cataract in a man æt. 74 years. After the operation, prescribed a four-grain solution of atropia, to be used by instillation into the eye. Previously, had prescribed ¼ grain doses of morphia, to be taken in conjunction with a cough mixture for bronchitis. During the night the patient took dr. j of the atropia solution in mistake for the morphia mixture. The characteristic toxicological effects developed, as great heat and burning in the throat, swelling of the tongue, numbness of the limbs, loss of consciousness, &c. Administered ¼-grain doses of morphia; after which the patient began to improve, and by four o'clock the next afternoon the unpleasant symptoms produced by the atropia had entirely disappeared.

---

*Smoking Belladonna in Asthma.*—Dr. Reeves, in the *Melbourne Medical Record*, states that smoke from the leaves of belladonna possesses much more power in cutting short an attack of asthma than that from stramonium. A long pipe is the best means of smoking them, the patient being instructed to draw the smoke deep into the chest. If when the attack is at its height he has not the power of doing this, the leaves may be placed in a saucer containing lighted charcoal or wood-ashes, which should be placed on a chair in front of the patient, this chair, as well as his own, being covered with a large sheet, so as to confine the fumes, before the leaves are put on the hot charcoal. From two and a half to five grains of the leaves are sufficient when smoked, and from five to twenty grains when burned.

---

*Emulsion of Phosphorated Oil.*—Prof. Redwood recommends the following method of exhibiting phosphorous in solution: Take one drachm of phosphorated oil, two drachms of yolk of egg, six drachms of syrup of tolu, and sufficient chloroform water to make six ounces and seven drachms. Make an emulsion, and add one drachm of liquor potassæ. The chloroform is introduced simply to preserve the preparation.—*Detroit Rev. Med.*

*A Simple Method of Treating Umbilical Hernia in Infants.*—A piece of white wax is softened, and fashioned with the fingers into a ball, which is then cut in two, so as to form two hemispheres. One of these hemispheres, which must be of a size proportionate to the umbilical ring, is applied to it in such a way that its spherical surface securely fills the opening, and is then retained in position by a strip of plaster. Instead of wax we may use gutta-percha, previously softened in warm water. Both of of these substances, about two hours after their application, become sufficiently softened to the skin. If the plaster excites cutaneous erythema, it should be removed every two days, and the skin powdered with rice-powder.—*Le Bordeaux Medical,* September 12th.

---

*Radical Cure for Piles.*—Dr. A. B. Bowen, of Magnoketa, Iowa, writes: "In a recent number of *The Record*, my attention was directed to the treatment for *nævus* by hypodermic injection. From the similarity of the anatomical structure of the nævus to hemorrhoidal tumors, I was induced to try the remedy. In the latter I used carbolic acid and ergot (fluid extract) in equal parts, injecting from ten to fifteen minims of the solution into the spongy, vascular hemorrhoidal tumor. This was repeated about once a week for five or six times, when the tumor had entirely disappeared. I have tried this in several cases, and it acts like a specific."—*Pacific Med. and Surg. Jour.*

---

*The Medical Uses of Myrrh.*—M. Deliaux de Savignac, considers its action upon the stomach as being well established. He has seen painful dyspepsia rapidly ameliorated under its use. It acts as a general tonic in gastralgia dependent upon several chronic diseases, anæmia and chlorosis especially. Finally, its local calmative and disinfectant action makes it a useful addition to certain topical applications. The following formulæ are recommended, by the author referred to, in the *Dictionaire encyclopedique des sciences medicales :*

R. Prepared chalk........500 grammes.
Powdered borax......250 "
Myrrh..............125 "
Orris root...............125 "
M.

Antigastralgic wine of myrrh, prescribed in the dose of a wine-glassful, three times a day, before or after meals, according to the time at which the pain is experienced, has an excellent stomachic effect; it is prepared as follows:

R. Best myrrh, powd. 20 grammes dr.v.
Bitter orange peel....15 " dr.iv.
Malaga wine.......... 1 litre f.oz.xxviii.

Macerate ten days and filter.—*Jour. de Med. et de Chir. partiques,* Nov. 1876.—*Clin. Rec.*

---

*Pilocarpin.*—A substance has been separated from the leaves of jaborandi (*pilocarpus pinnatus*) that seems to possess all their virtues; it has been called *pilocarpin.* It has a semi-fluid existence, has a yellowish color, an agreeable odor, is free from acidity, and is spoken of as an alkaloid. In doses of one-twelfth to one-fourth of a grain, it is equal to an infusion containing the strength of from five to ten grains of the leaves. It has but little effect on the heart's action, or on the temperature of the body, nor does it act as a narcotic on the brain. Jaborandi is in truth a harmless but highly potent medicine, and its efficacy is in its action as a sialagogue and diaphoretic.—*Druggist's Circular.*

*The Sudden Checking of Opium Eating.* The eminent Sir Robert Christison, after a large experience in the treatment of such cases, says that no good can be done by "gradual reduction," and that it can be safely left off abruptly, even after many years' indulgence. He recommends bromide of potassium to allay irritability, and chloral to procure sleep. For the first three days the patient suffers from great depression, loathing, sickness and vomiting. By the fourth night he falls asleep and awakes refreshed, and in most cases the progress afterward is very satisfactory. There is, however, great danger of a relapse. Should diarrhœa supervene, suppositories of morphia should be ordered.

*The Use of Conium.*—Dr. A. M. Hamilton says that in the treatment of diseases where tremor is a symptom, much benefit has followed the use of conium at the female epileptic and paralytic hospital on Blackwell's Island. In two cases of chorea of long standing it produced a prompt amelioration of the patients' condition, and in the tremor sclerosis it suppressed the movements for several weeks. It was given in the form of fluid extract, in doses of min. x three times a day.—*Med. and Surg. Reporter.*

*Chloral in Ulcers.*—Dr. W. M. Wright, Surgeon and Secretary of the National Home for Disabled Volunteer Soldiers (Southern Branch), Hampton, Va., writes us:

"I find nothing so valuable in the treatment of old indolent ulcers, not dependent upon necrosis, as a local application of a solution of hydrate of chloral. Among the old soldiers of our home we have a great variety of such cases, and I find nothing improves their condition better or more rapidly than the above."

*Prevention of After-Pains.*—Dr. Le Diberder (*Ann. de Gynecolog.*) believes that ergot, suitably administered, has the power of preventing after-pains. He gives half a drachm in divided doses directly after the expulsion of the placenta, with the object of bringing about a firm and consistent contraction of the uterus in place of the alternate contractions and relaxations to which he says after-pains are dne. The *Dublin Med. Press and Circ.*, in commenting upon this statement, calls attention to the opinion of Sir Charles Locock, that after-pains were due to the retention of coagula, and that firm manual pressure upon the uterus to promote their expulsion was never followed by after-pains.

*Creasote by Spray.*—Commence with a weak solution of creasote, two minims to the ounce of water, and gradually increase it to twice that strength. A sufficient quantity of spirit should be added to dissolve the creasote. I direct the patient to take one deep inspiration, so as to entice the spray well into the lungs, and again to renew it in the course of a few seconds. After one or two applications it gives rise to no irritation or cough, and it is extremely agreeable to the patient.

*Castor Oil.*—This nauseous drug can be easily taken when administered in the following way: The glass should be first rinsed with moderately hot water, and then two drachms of hot water, one ounce of castor oil and one drachm of peppermint water put in the order mentioned. This is easily swallowed, and leaves hardly any taste of the oil in the mouth.

*Vegetable Diet for Epileptics.*—Dr. Merson concludes that there are fair grounds for the deduction that farinaceous food is more suitable for epileptics than a mixed or nitrogenous diet.

*Valuable Prescriptions.*—Dr. Vanderbeck, in *Med. and Surg. Rep.*, publishes the following formulæ in chemical and hospital practice in New York:

EPILEPTIC SEIZURES AT THE MENSTRUAL PERIOD.

The case in question was a young woman who menstruated only once in six weeks, and then the flow was very scanty. The convulsions were pronounced to be due to reflex irritation, from congestion of the ovaries.

R. Aloes,.................... gr.j.

Belladonnæ ext....... gr.$\frac{1}{6}$.

Capsici, ................. gr.$\frac{1}{4}$.

F. pil.

Sig.—Taken every evening, for a few days before menstrual period. Just at this time, leeches, applied over the ovaries, and warm baths, will be of service. The diet must be of easy digestibility.

Also use the following prescription:

R. Pot. brom., ........ grs. xx.
Tinct. belladon...... min. ij.
Syrupi,
Aquæ, aa q.s. ad. ft. dr ij.

Sig.—One dose, three times a day.

It may be remembered that it was in just such cases as these, of convulsions attending disorders of menstruation, bromide of potash first came into use. It was soon discovered that its antispasmodic virtue extended to all forms of epileptic seizures, whether connected with some obvious irritation, or having no such dependence, being idiopathic in character.

SUPRA-ORBITAL NEURALGIA (*Syphilitic*).

R. Pot. iod., gr.x.

One dose, ter die, in solution, after meal. Increase the dose to twenty grains, after a time.

Also—

R. Ung. aconitæ, strength, gr. ss, to adipis oz j.

Sig—Rub over painful part.

UNCOMPLICATED SUPRA-ORBITAL NEURALGIA.

R. Arsenici, .................. gr $\frac{1}{24}$

Ext. Conii, ............... gr j.

Ext. cannabis ind. ...... gr $\frac{1}{8}$.

Sig.—One dose, ter die. *Da Costa.*

OBSTINATE NEURALGIA.

R. Sodæ arseniatis,......... gr $\frac{1}{30}$

Cinch. sulph............. grs ij.

Conii ext. ................... gr j.

Sig.—One dose, ter die. During the paroxysm use hypodermic injection of morphia.

CEREBRAL NEURALGIA.

R. Chloral hyd............. grs x.
Pot. bromid............. grs xx.
Syr. aurant. cort. ...... dr ss.
Aquæ......................... dr iss. M.

Sig.—One dose, at bed time.

Also—

R. Tinc. cinch, comp....... oz ij.
Fl. ext. cinch............ oz j.
Ammon. brom............ oz ss. M.

Sig.—One teaspoonful, ter die.

UTERINE NEURALGIA.

R. Tinc. aconit. rad...... dr jss.
Ammon. chloridi...... dr ij.
Ammon. iod. ......... dr j.
Tinct. card. co......... oz j.
Syr. aurant cort........ oz iv.
Aq. anisi., q s. ad. ft... oz viij. M.

Sig.—One drachm, every four hours.

Also—

R. Syr. ferri. quiniæ et strichniæ phos.

Sig.—One drachm, half an hour before each meal.

OVARIAN NEURALGIA.

R. Ammon. mur............ dr ij.
Tinct. aconit ........... dr ij.
Syr. aurant. cort........ oz viij. M.

Sig.—One drachm, ter die. *Da Costa.*

---

—Dr. Roth related at the Medical Society of Strasburg, the case of a child three years old who vomited a button two years after it had been swallowed.

### Scientific Items.

The *bisulphide* is regarded as a very superior disinfectant for sick rooms, etc. When ignited, it evolves sulphurious acid vapor. It may be burned in a spirit lamp, or in an open vessel. It is highly inflammable, and should be cautiously handled.

*The Sun* is a molten mass, white hot, 856,000 miles in diameter, equaling the bulk of 1,260,000 worlds like the earth, having a surrounding ocean of gas on fire 50,000 miles deep, tongues of flame darting up 75,000 miles, volcanic forces that hurl luminous matter to the height of 160,000 miles. He travels, also, in an orbit which requires a period of 18,-000,000 of years! The spectroscope reveals the existence on the sun of all the metals that are found in the earth

*Meteorological Observations.*—In 1873, the Meteorological Congress at Vienna proposed that at least one observation should be taken, at a given time, at every signal station throughout the world. The Bureau at Washington has accordingly perfected an arrangement by which, at the hour of 7:35 A. M., observations will be taken in the United States, Great Britain, Austria, Algeria, Belgium, Denmark, France, Germany, The Netherlands, Norway, Russia, Portugal, Sweden, Spain, Switzerland, Greece, Turkey, Canada, Hawaiian Islands, Dutch Guinea and Japan, and upon all the naval vessels of the United States.

*Venous Pulse Resulting from Chloroform.*—Prof. Leon Noel, of the University of Louvain, has published an article in a French journal, in which he announces as a symptom of the physiological action of chloroform the fact that the internal jugular veins, the subclavian, and, in most cases, the external jugular, and sometimes even the facial veins, are the seat of pulsations which are isochronous with the radial pulse. These pulsations are scarcely sensible to the touch, but very perceptible to the eye. The Professor thinks that a venous pulse so marked as this, indicates a profound perturbation of the functions of the heart.

*The Microscope.*—In the animalcule world the law of devourer and prey holds good. In a single drop of water the larger infusoria have been seen to pursue and devour the smaller ones. In the language of Hudibras—

> "These fleas have other fleas to bite 'em,
> And these fleas fleas ad infinitum."

*Œnothera Biennis.*—For that form of asthma which at certain seasons manifests itself, particularly at night, and associated with bronchial irritation and grastric disturbance, flatulency, etc., Dr. Davis, in the *American Practitioner*, says that he has found the *Œnothera Biennis*, or evening primrose, to be a valuable remedy. Thinks it has a specific action upon the pneumogastric nerve, and is adapted to the treatment of such cases of respiratory or gastric trouble as involve a morbid sensitiveness either in the laryngeal, pulmonary, or gastric branches of that nerve. He gives the fluid extract in doses of twenty-five drops in half tablespoonful of water before each meal and at bed time.

*Value of a Finger.*—A court in New York has established the value of a human diget, by giving a laborer, who had his finger mashed in moving a barrel of lard, damages against his employer to the amount of $1,000.

### Practical Hints.

*Eczena.*—In a clinical lecture by Dr. Yandell, in *Louisville Medical News*, he remarks:—Quinia is the remedy for acute eczena in a vast majority of cases. Arsenic and other antiperiodics may substitute it. Iron is always needed.—Calomel in cathartic doses promotes recovery. Local taeatment is not with–out benefit. These children will get the following prescriptions:

Tannin,........................gr. x;
Morphia,........................gr. ij;
Carbolic Acid,............gr. ij;
Benzoated oxide of zinc ointment (any other unirritating ointment might do as well),................1 ounce.

Mix thoroughly and apply to the eruption. No soap must be used, and washing, even with simple water, should be done as seldom as possible.

Calomel.....................gr. x;
Bicarb. Soda................gr. l.

Mix and make ten powders. Give one thrice a week at bed-time.

Sulphate of Quinia......1 drachm.
Tannin,...... ... ...........gr. xv;
Syrup of tolu,...............ounces iiij.

Mix carefully. Direct to shake well before administering, and give each child fou teaspoonsful daily, the last to be taken two hours before the period of severe itching is expected to commence. The first dose is to be given four to six hours preceeding the last. Properly compounded this is a tasteless mixture and therefore excellent for children.—It is readily absorbed, It seldom nauseates. One of these children is two and a half years old; the other is a year younger. Children bear and require larger doses of quinia in proportion than adults. The antiperiodic treatment will be followed by the ferruginous and bitter tonics. As to diet, the children should have whatever they will eat.—Meat and fruits are especially good for them. Never put on low diet any of your patients with skin diseases, and encourage all to use fats.

*Rheumatism Treated with Salicylate of Soda.*—Professor A. Clarke.—Prof. C. treated eleven cases of acute rheumatism —all that occurred in his ward of Belle. vue from April 1 to June 1—with thi drug. In nine of the cases there was early improvement following the use of the medicine. In two cases the amelioration was more gradual. The influence of the medicine in "lowering the fever heat and diminishing the excited pulse were as marked as its power to relieve pain."

The formula used in all the cases is as follows:

R—Acid salicylic.............. .....dr. iij;
Sodæ bicarbonat.. ..............dr. ij;
Glycerine, Aq. } *aa* ................ ...oz. ij

M. Sig. Tablespoonful every two hours for the first day, and afterward the same dose six times a day.

No unpleasant effect of any kind was noticed after the administration of the medicine.—*Med. Record, Oct.* 14, 1876

"*Important, if True.*"—An Englishman of some note sends to a Liverpool paper the remarkable statement that the worst case of small-pox can be cured in three days, simply by the use of cream of tartar. One ounce of cream of tartar dissolved in a pint of water, drank at intervals, when cold, he says, is a certain remedy; it has cured thousands, never leaves a mark, never causes blindness, and avoids tedious lingering.

